# Helical computed tomography scanning of the larynx and upper trachea in rabbits

**DOI:** 10.1186/s13028-015-0157-4

**Published:** 2015-10-01

**Authors:** Amr M. Ajlan, Talal Al-Khatib, Mariam Al-Sheikah, Saddig Jastaniah, Alamin Salih, Abdulrahman Althubaiti, Abdulrahman Aljohani, Hani Marzouki, Ameen Alherabi, Osama Marglani, Samar Rabah, Gamal Karrouf

**Affiliations:** Radiology Department, King Abdulaziz University Hospital, King Abdulaziz University, Jeddah, Western Region Saudi Arabia; Department of Otolaryngology, Head and Neck Surgery, Faculty of Medicine, King Abdulaziz University, Jeddah, Saudi Arabia; Department of Diagnostic Radiology, Faculty of Applied Medical Science, King Abdulaziz University, Jeddah, Saudi Arabia; Department of Otolaryngology, Head and Neck Surgery, Umm Al-Qura University, Mecca, Saudi Arabia; Department of Biology, Faculty of Science, King Abdulaziz University, Jeddah, Saudi Arabia; Experimental Surgery Unit, King Fahd Medical Research Center, King Abdulaziz University, Jeddah, 21589 Saudi Arabia; Department of Surgery, Anesthesiology and Radiology, Faculty of Veterinary Medicine, Mansoura University, Mansoura, 35516 Egypt

**Keywords:** Helical computed tomography, Trachea, Subglottis, Rabbits

## Abstract

**Background:**

Computed tomography (CT) is used to evaluate the human tracheobronchial tree because of its unsurpassed ability to visualize the airway and surrounding structures. To establish an ideal animal model for studying subglottic stenosis, we assessed the size and morphology of the normal rabbit’s laryngotracheal airway by helical CT. We measured luminal dimensions at the levels of the arytenoid and cricoid cartilages and the first, third, and eighth tracheal rings. At all levels, the axial slices were used to calculate the maximum anteroposterior (AP) dimension, transverse dimension, and cross-sectional areas. We measured the tracheal length from the cricoid to the third and eighth tracheal rings on sagittal reformation. We assessed the hyoid, thyroid, cricoid, arytenoid, and tracheal rings for the presence of calcific or soft tissue densities. We also addressed the presence or absence of pre-epiglottic and paraglottic fat.

**Results:**

The mean AP tracheal dimension ± standard deviation (SD) was 8.6 ± 0.5 mm at the arytenoid level, 8.2 ± 0.7 mm at the cricoid level, and 7.7 ± 0.2 mm at the first tracheal ring level. The transverse tracheal dimension ±SD was 5.3 ± 0.1 mm at the arytenoid level, 5.5 ± 0.5 mm at the cricoid level, and 6.1 ± 0.6 mm at the first tracheal ring level. The mean tracheal area ±SD was 35.7 ± 2.2 mm^2^ at the arytenoid level, 35.8 ± 5.1 mm^2^ at the cricoid level, and 39.2 ± 4.3 mm^2^ at the first tracheal ring level. The tracheal length ±SD was 10.7 ± 2.3 mm from the cricoid to the third tracheal ring and 19.1 ± 1.14 mm to the eighth tracheal ring. There was complete calcification of the hyoid in all rabbits. Only two rabbits showed complete thyroid, arytenoid, or tracheal ring calcification. The remaining airway components were otherwise either uncalcified or partially calcified. The uvula, epiglottis, aryepiglottic fold, vallecula, piriform sinus, true/false vocal cords, and pre-epiglottic/paraglottic fat were not seen in any rabbit.

**Conclusions:**

Helical CT investigation provides good, highly definitive anatomic details of the larynx and trachea in rabbits. Such results may be used in further evaluation of the normal airway and in cases of subglottic stenosis.

## Background

Imaging-based anatomical studies are of great importance in biomedical research because they constitute a bridge between preclinical assessment of normal anatomy and clinical investigation of disease status. Computed tomography (CT) provides detailed anatomical images [1] and is used as a valuable noninvasive tool in the evaluation of the human laryngotracheobronchial tree. The role of CT has been proven useful in the assessment of focal and diffuse neoplastic and non-neoplastic airway abnormalities [2, 3].

The rabbit’s airway is a reliable and reproducible model for the study of subglottic stenosis because rabbits tolerate interventions with minimal morbidity and mortality and their larynx roughly approximates the size of that of children. Thus, the rabbit model has been used extensively to study the subglottic airway [4–8]. The standard size and anatomical characteristics of the rabbit’s larynx and trachea have been studied using anatomical cross sections [9]. To the best of our knowledge, however, examination of the normal rabbit’s airway by CT has not been reported. Thus, we aimed to obtain baseline CT data by evaluating the dimensions and morphological characteristics of the larynx and upper trachea in healthy rabbits.

## Methods

### Animals

Four mature clinically healthy male New Zealand white rabbits were used. The rabbits were 12 months of age, weighed 3.8–4.5 kg, and were housed at 25 °C with a 12-h dark/light cycle. They were allowed several days to acclimatize to their cages and surroundings [9]. Each animal was anesthetized using a combination of intramuscularly administered 50-mg/kg ketamine (Tekam 50; Hikma Pharmaceuticals, Amman, Jordan) and 5-mg/kg xylazine (Xyla-Ject; ADWIA Pharmaceuticals, El Obour, Egypt) [10].

All studies were carried out in the Experimental Surgery Unit at King Fahd Medical Research Center, King Abdulaziz University (KAU). The CT scans were performed at the Radiology Department, Faculty of Applied Medical Science, KAU. The protocol met the approval of the Institutional Animal Care and Use Committee at KAU. The study was performed in strict compliance with the ethical guidelines for humane treatment of animals as defined by KAU.

### CT scanning procedures

Helical CT was performed using a 16-detector row scanner (BrightSpeed S; General Electric, Milwaukee, WI, USA). Imaging was performed from the mandible to the clavicle in the craniocaudal direction while the rabbit’s neck was in full extension. The following parameters were applied: collimation of 16 × 0.625 (1.25–3.75) mm, pitch of 3, gantry rotation time of 280 ms, tube voltage of 90 kV, and tube current–time product of 10 mA(s). All images were transferred to an external Digital Imaging and Communications in Medicine (DICOM) reader (OsiriX, version 6.5; Pixmeo, Bernex, Switzerland) in transverse and sagittal reformats and assessed in the soft tissue window.

Several laryngotracheal morphological features were evaluated. The hyoid, thyroid, cricoid, arytenoid, and tracheal cartilages were assessed for the presence of calcified or soft tissue densities. When a calcified density was observed, it was characterized as partial or complete. The level of the thyroid cartilage in relation to the cervical spine was determined. The shape of the tracheal lumen was also assessed at the hyoid and mid-tracheal levels. The ability to distinguish the uvula, epiglottis, aryepiglottic fold, vallecula, piriform sinus, true vocal cord, and false cord was assessed. The presence of pre-epiglottic and paraglottic fat was noted as well.

**Fig. 1:**
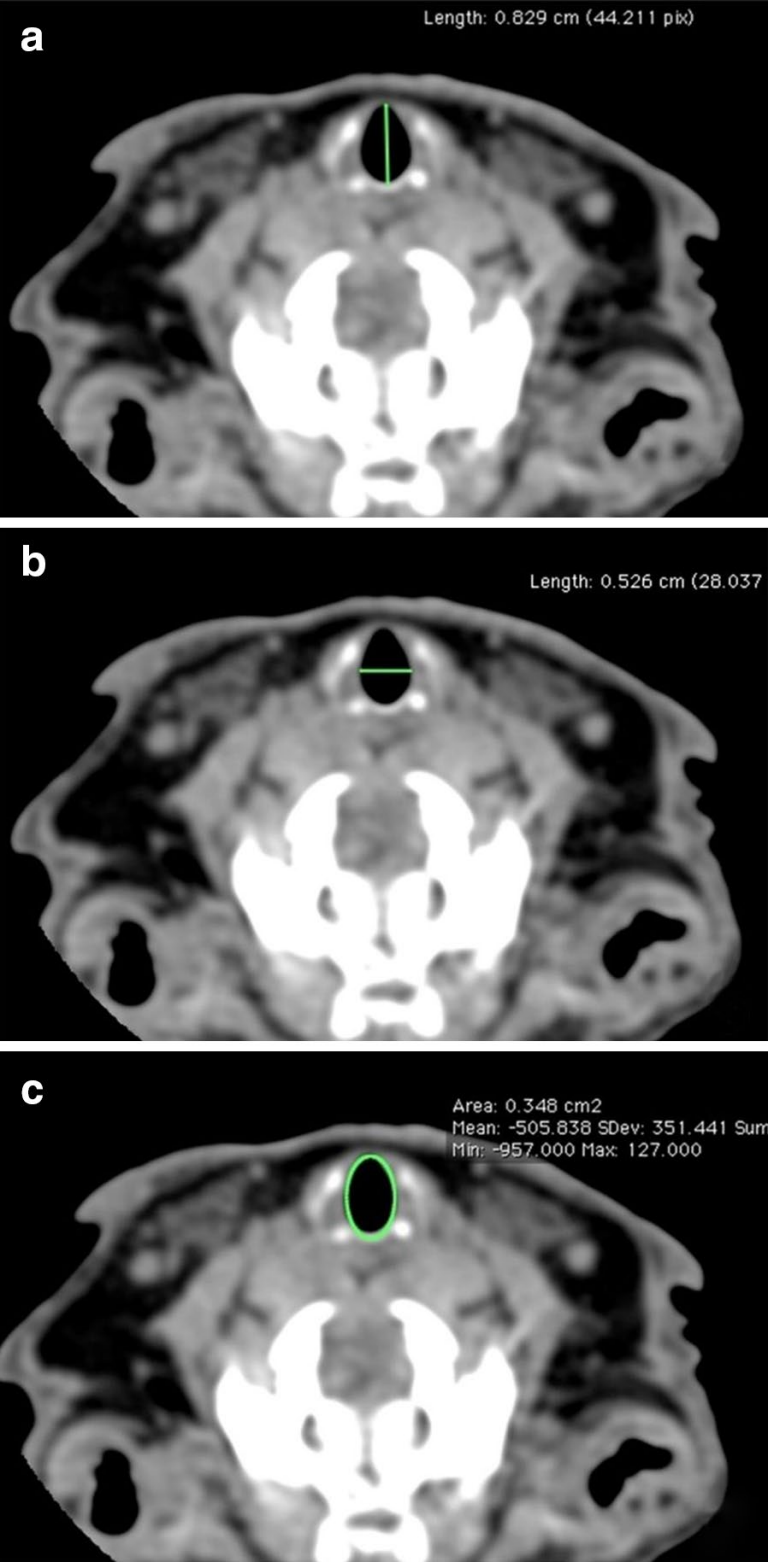
Representative images of computed tomography measurements used in this study. **a** Transverse CT image showing the trachea with an anteroposterior diameter of 8.3 mm at the arytenoid cartilage level. **b** Transverse CT image showing the trachea with a transverse diameter of 5.3 mm at the arytenoid cartilage level. **c** Transverse CT image showing the trachea with an area of 34.6 mm^2^ at the arytenoid cartilage level

An experienced radiologist performed the tracheal measurements manually at the levels of the arytenoid, cricoid, first tracheal ring, third tracheal ring, and eighth tracheal ring. At all levels, the axial slices were used to calculate the maximum anteroposterior (AP) diameter, transverse diameter, and cross-sectional area (Fig. 1a–c). The cursors were placed on the inner walls of the trachea for all lumen measurements. The tracheal length from the cricoid to the third and eighth tracheal rings was measured on the sagittal reformation (Fig. 1d).

### Statistical analysis

Data are presented as mean ± standard deviation (SD). Data from the five tracheal regions (arytenoid, cricoid, first tracheal ring, third tracheal ring, and eighth tracheal ring levels) were analyzed using nonparametric one-way analysis of variance followed by the Bonferroni multiple range test (IBM SPSS software; Chicago, IL, USA). Differences were considered significant at *P* < 0.05.

## Results

**Fig. 2:**
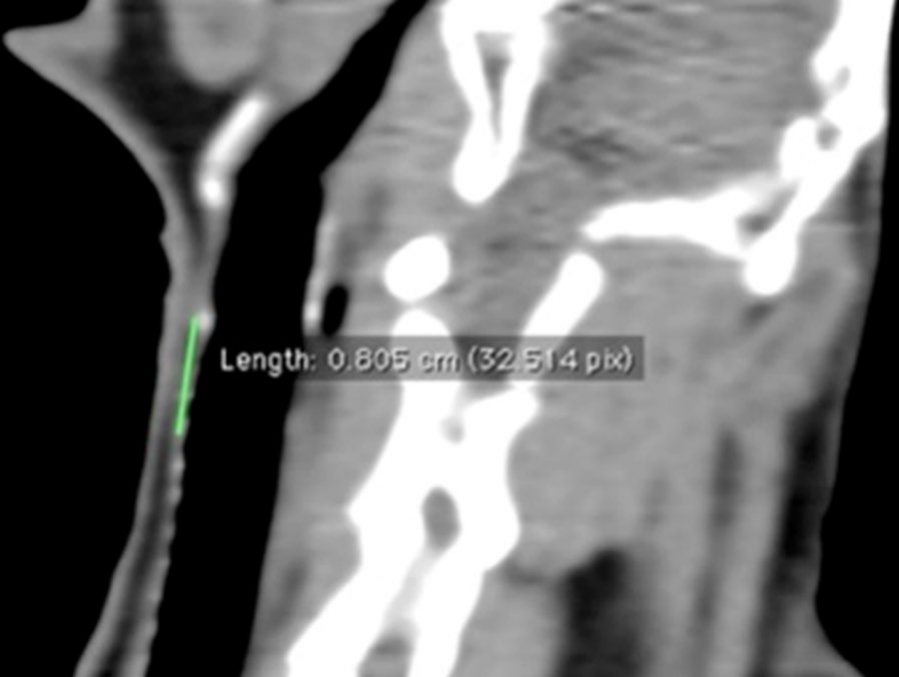
Sagittal CT image showing a distance of 8.1 mm from the cricoid cartilage to the third tracheal ring

The tracheal lumen measurements are summarized in Table 1, and examples are presented in Figs. 1 and 2. The mean AP luminal tracheal dimension ±SD was 8.6 ± 0.5 mm at the arytenoid level, 8.2 ± 0.7 mm at the cricoid level, 7.7 ± 0.2 mm at the first tracheal ring level, 6.9 ± 0.3 mm at the third tracheal ring level, and 6.8 ± 0.3 mm at the eighth tracheal ring level. The mean luminal transverse tracheal dimension ±SD was 5.3 ± 0.1 mm at the arytenoid level, 5.5 ± 0.5 mm at the cricoid level, 6.1 ± 0.6 mm at the first tracheal ring level, 6.6 ± 0.2 mm at the third tracheal ring level, and 7.3 ± 0.1 mm at the eighth tracheal ring level. The mean luminal tracheal area ±SD was 35.7 ± 2.2 mm^2^ at the arytenoid level, 35.8 ± 5.1 mm^2^ at the cricoid level, 39.2 ± 4.3 mm^2^ at the first tracheal ring level, 36.5 ± 2.1 mm^2^ at the third tracheal ring level, and 4.2 ± 3.3 mm^2^ at the eighth tracheal ring level. The tracheal length ± SD was 10.7 ± 2.3 mm from the cricoid to the third tracheal ring and 19.1 ± 1.14 mm from the cricoid to the eighth tracheal ring.

**Table 1:** Laryngeal and tracheal measurements in four rabbits at different locations of the larynx and trachea

Structure	Measurements	Rabbit no.				Mean	Standard deviation
		1	2	3	4		
Arytenoid	Anteroposterior (mm)	9.2	8.2	8.8	8.1	8.6	0.5
	Transverse (mm)	5.3	5.3	5.3	5.4	5.3	0.1
	Surface area (mm^2^)	37.6	34.6	37.4	33.1	35.7	2.2
Cricoid	Anteroposterior (mm)	7.6	7.6	8.7	8.9	8.2	0.7
	Transverse (mm)	5.6	4.8	6.0	5.6	5.5	0.5
	Surface area (mm^2^)	34.2	30.3	42.6	36	35.8	5.1
1st tracheal ring	Anteroposterior (mm)	7.6	7.6	7.8	7.9	7.7	0.2
	Transverse (mm)	5.4	5.9	6.7	6.3	6.1	0.6
	Surface area (mm^2^)	33.9	37.5	43	42.3	39.2	4.3
3rd tracheal ring	Anteroposterior (mm)	6.5	7.1	7	7.1	6.9	0.3
	Transverse (mm)	6.3	6.5	6.8	6.8	6.6	0.2
	Surface area (mm^2^)	33.5	36.7	37.5	38.3	36.5	2.1
8th tracheal ring	Anteroposterior (mm)	6.8	6.9	7.2	6.4	6.8	0.3
	Transverse (mm)	7.3	7.2	7.4	7.2	7.3	0.1
	Surface area (mm^2^)	40.3	38.9	44.7	36.8	40.2	3.3
Cricoid-3rd tracheal ring	Length (mm)	9.5	8.1	12.8	12.4	10.7	2.3
Cricoid-8th tracheal ring	Length (mm)	22	24.6	26.9	22.7	19.1	1.14

**Fig. 3:**
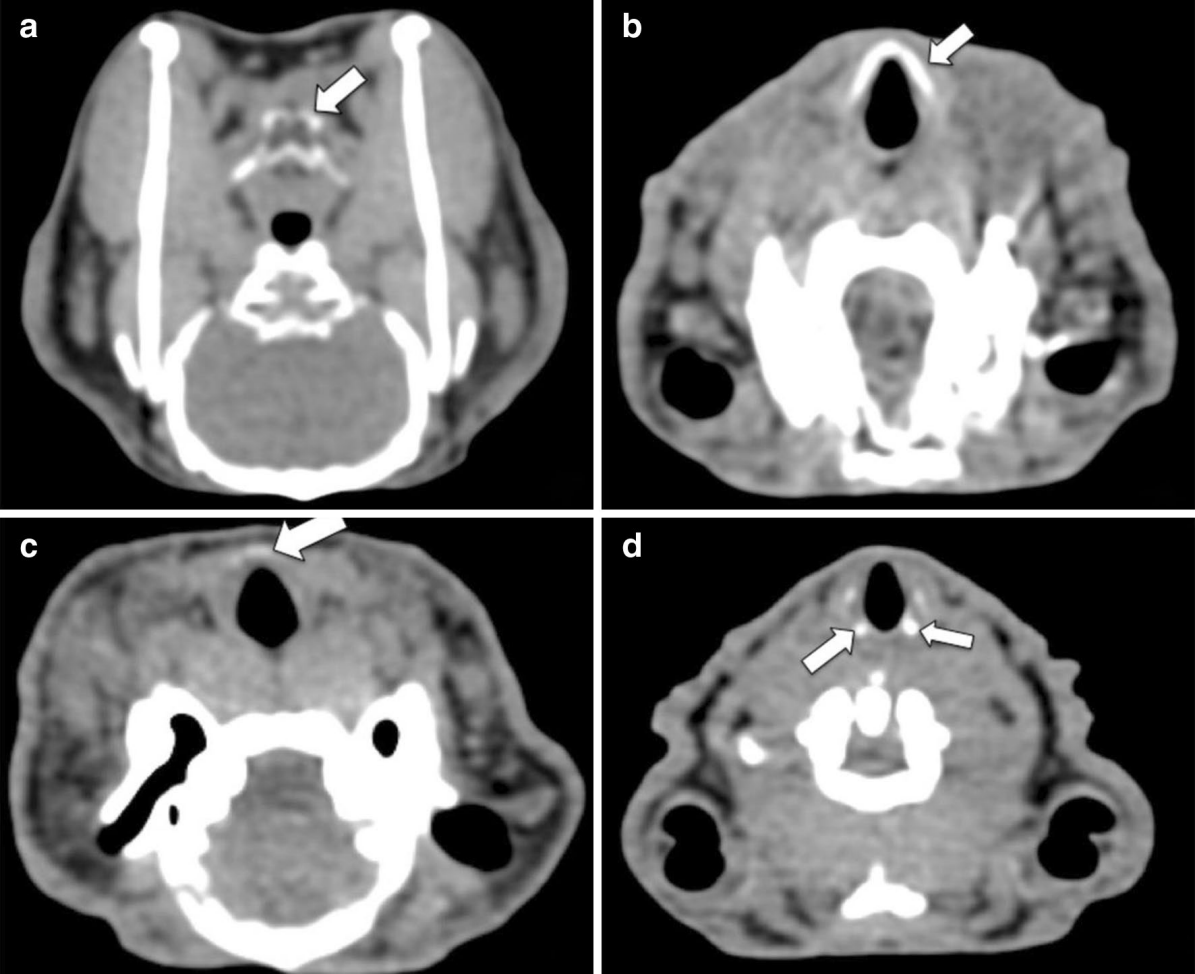
Representative images of various computed tomographic morphological appearances of the rabbit’s laryngeal components. **a** Diffusely and densely calcified hyoid cartilage (*arrow*). **b** Diffusely and densely calcified thyroid cartilage (*arrow*). **c** Partial and faint thyroid cartilage calcification (*arrow*). **d** Densely calcified arytenoid cartilage (*arrows*)

The morphological features of each rabbit are summarized in Table 2. The thyroid cartilage was located at the level of the first cervical vertebral body in all rabbits. The lumen of the larynx at the hyoid level was diamond-shaped in all but one rabbit, which had an oval-shaped lumen. The lumen appeared nearly rounded in all rabbits at the mid-tracheal level. The hyoid cartilage was completely and densely calcified in all rabbits (Fig. 3a), and the articulation between the hyoid body and horns was readily distinguishable. The thyroid cartilage was completely and densely calcified in two rabbits (Fig. 3b) and had faint partial calcifications in the other two (Fig. 3c). Two rabbits had dense arytenoid calcifications (Fig. 3d), while the other two had arytenoids of soft tissue density. The cricoid cartilage calcifications were complete in two rabbits and partial in the other two. All tracheal rings were completely calcified in two rabbits, partially calcified in one rabbit, and of soft tissue density in one rabbit (Table 2).

**Table 2:** Body weights and computed tomographic morphologic findings of the larynx and trachea of the four rabbits in this study

Rabbit no.	Weight (kg)	Hyoid calcification	Hyoid body-horn articulation	Thyroid calcification	Arytenoid calcification	Cricoid calcification	Tracheal rings calcification	Pyriform sinus
1	3.8	Complete and dense	Easily distinguishable	Complete	Complete	Partial	All rings completely calcified	None
2	4.0	Complete and dense	Easily distinguishable	Complete	Complete	Partial	All rings completely calcified	None
3	4.5	Complete and dense	Easily distinguishable	Partial	None	None	None	None
4	4.8	Complete and dense	Easily distinguishable	Partial	None	Partial	All rings partially calcified	Air-filled on left side

**Fig. 4:**
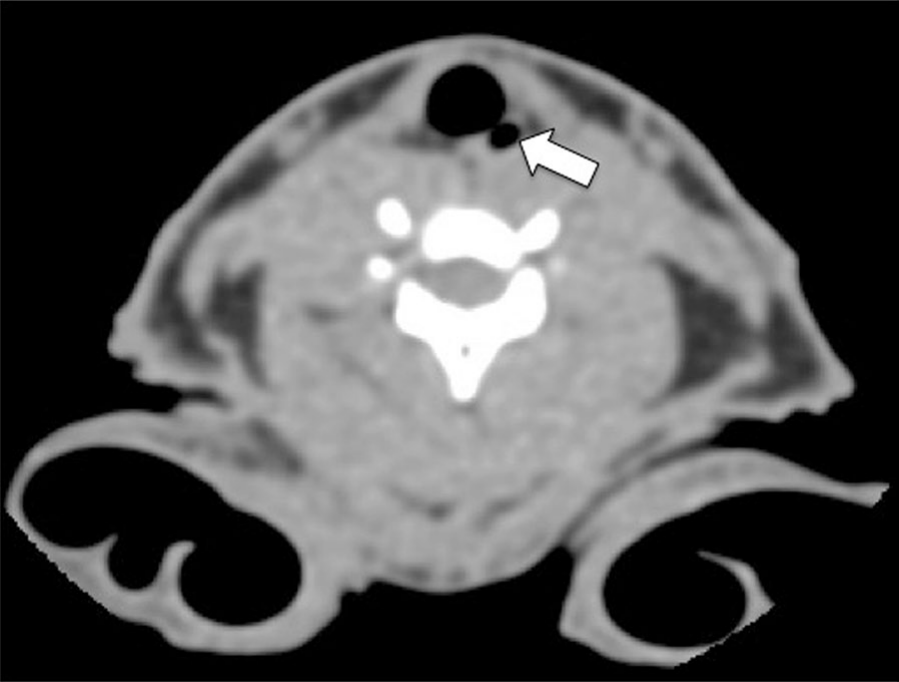
CT image of a rabbit’s airway showing an aerated left piriform sinus (*arrow*)

One rabbit showed a small air-filled left piriform sinus (Fig. 4). The piriform sinus was not seen in any of the other rabbits. Additionally, the uvula, epiglottis, aryepiglottic fold, vallecula, piriform sinus, true vocal cords, false cords, pre-epiglottic fat, and paraglottic fat were not seen in any of the rabbits.

## Discussion

The effect of various pathologies on the tracheal size and morphology is an important clinical feature that may affect management decisions [3, 4]. The degree of stenosis cannot be ascertained with accuracy without reference to normal baseline tracheal measurements. Although our results provide baseline measurements of the normal rabbit’s trachea, such data have the potential to be extrapolated to the human airway. Several authors have used the normal and abnormal rabbit airway as a model to better understand laryngotracheal diseases in humans [9, 12].

To the best of our knowledge, the normal morphology of the rabbit’s larynx and trachea has not been previously described by CT. Use of rabbit models to correlate the normal rabbit’s larynx and tracheal morphology to that of the normal pediatric human airway may help to understand human pediatric laryngotracheal disease [13]. The articulation between the hyoid body and horn could be easily seen in the calcified hyoid cartilage. This articulation is also easily seen in humans. The thyroid cartilage is typically dense in humans, while we found that some rabbits had partially and fainter thyroid cartilage density. Our study also showed that the laryngeal cartilage of rabbits might have denser calcification than that of humans [1, 3].

We performed helical CT scans of the rabbit subglottis and trachea in dorsal recumbency. This approach conceivably allowed more detailed structural imaging assessment of the rabbits’ subglottis. The mean AP and transverse tracheal diameter at the cricoid level was 5.8 ± 0.45 and 5.4 ± 0.39 mm, respectively. In the same group of rabbits, the mean AP and transverse tracheal diameter at the eighth tracheal cartilage level was 4.7 ± 0.48 and 5.9 ± 0.59 mm, respectively. Loewen and Walner [9] found no significant variations in tracheal dimensions despite weight ranges from 2.3 to 5.1 kg. Our rabbits had an average transverse diameter of similar size at the cricoid level, but the average diameter at the other levels of all other cricoid and the eighth tracheal ring was larger. The reason for this variation is not clear; it may have been because of differences in the size of the rabbits or measurement techniques between the gross anatomical dissection specimens and CT images.

The mean rabbit tracheal AP dimension corresponded closely to that of 2- to 8-year-old children, the age at which the average AP tracheal diameter ranges from 7.4 ± 0.8 to 9.2 ± 1.1 mm. Children 0–4 years old have transverselateral tracheal dimensions of 6.4 ± 1.2 to 8.1 ± 0.7 mm, which resembles the rabbit tracheal dimensions found in this study. The tracheal area of children aged 0–4 years closely resembles that in our rabbits (28 ± 9 to 48 ± 8 mm^2^) [14].

It was difficult to appreciate the uvula, epiglottis, aryepiglottic fold, vallecula, piriform sinus, true vocal cords, and false cords on CT in our rabbit model. Only a single rabbit showed an aerated unilateral piriform sinus. Such structures are more easily seen on CT images of the human neck. A feature similar to human anatomy is the absence of pre-epiglottic and paraglottic fat on CT [15].

Our results support the hypothesis of Fishman et al. [15], who suggested that helical CT is one of the best methods for non-invasive imaging of the airway. Our helical CT findings of the rabbit’s subglottis and trachea also correlate with the anatomical data of Loewen and Walner [9] and Zotti et al. [13] with respect to subglottic morphological features and tracheal measurements.

## Conclusion

Thin-slice helical CT of the normal rabbit’s larynx and trachea provides a convenient and useful noninvasive imaging method to assess tracheal size and morphology. The results may be used as a basis for further evaluation of the normal laryngotracheal airway and diagnostic assessment of subglottic stenosis.
